# Transcranial Laser Photobiomodulation Improves Intracellular Signaling Linked to Cell Survival, Memory and Glucose Metabolism in the Aged Brain: A Preliminary Study

**DOI:** 10.3389/fncel.2021.683127

**Published:** 2021-09-03

**Authors:** Fabrízio dos Santos Cardoso, Fernanda Cristina Borini Mansur, Rodrigo Álvaro Brandão Lopes-Martins, Francisco Gonzalez-Lima, Sérgio Gomes da Silva

**Affiliations:** ^1^Núcleo de Pesquisas Tecnológicas, Universidade de Mogi das Cruzes, Mogi das Cruzes, Brazil; ^2^Department of Psychology and Institute for Neuroscience, University of Texas at Austin, Austin, TX, United States; ^3^Hospital Israelita Albert Einstein, São Paulo, Brazil; ^4^Programa de Pós Graduação em Bioengenharia, Universidade Brasil, São Paulo, Brazil; ^5^Centro Universitário UNIFAMINAS (UNIFAMINAS), Muriaé, Brazil; ^6^Hospital do Câncer de Muriaé, Fundação Cristiano Varella (FCV), Muriaé, Brazil

**Keywords:** photobiomodulation, low-level laser therapy, brain, aging, intracellular signaling proteins

## Abstract

Aging is often accompanied by exacerbated activation of cell death-related signaling pathways and decreased energy metabolism. We hypothesized that transcranial near-infrared laser may increase intracellular signaling pathways beneficial to aging brains, such as those that regulate brain cell proliferation, apoptosis, and energy metabolism. To test this hypothesis, we investigated the expression and activation of intracellular signaling proteins in the cerebral cortex and hippocampus of aged rats (20 months old) treated with the transcranial near-infrared laser for 58 consecutive days. As compared to sham controls, transcranial laser treatment increased intracellular signaling proteins related to cell proliferation and cell survival, such as signal transducer and activator of transcription 3 (STAT3), extracellular signal-regulated protein kinase (ERK), c-Jun N-terminal kinase (JNK), p70 ribosomal protein S6 kinase (p70S6K) and protein kinase B (PKB), also known as Akt that is linked to glucose metabolism. In addition, ERK is linked to memory, while ERK and JNK signaling pathways regulate glucose metabolism. Specifically, the laser treatment caused the activation of STAT3, ERK, and JNK signaling proteins in the cerebral cortex. In the hippocampus, the laser treatment increased the expression of p70S6K and STAT3 and the activation of Akt. Taken together, the data support the hypothesis that transcranial laser photobiomodulation improves intracellular signaling pathways linked to cell survival, memory, and glucose metabolism in the brain of aged rats.

## Introduction

In the world, the number of people aged 60 and over has been growing faster than any age group (Desa, 2015). However, aging is accompanied by a cognitive impairment, which has been one of the socio-economic concerns in developed and developing countries (Attia and Ahmed, 2020). Among the various theories to interpret this damage, intracellular signaling proteins have gained special attention (Kosik et al., 2012). For example, the expression and activation of signaling proteins linked to cell growth, proliferation, and survival, as Akt, p70S6K and extracellular signal-regulated protein kinase (ERK), are decreased in the brain of aged rats, including the hippocampal formation, a brain region linked to certain mnemonic processes (Jin et al., 2008). Importantly, activation and expression of intracellular signaling proteins linked to apoptosis, such as c-Jun N-terminal kinase (JNK) and p38, are increased in the hippocampus of aged rats (O’Donnell et al., 2000). Current knowledge points to a crucial role of ERK and JNK signaling pathways in regulating glucose metabolism (Papa et al., 2019). In addition, Akt facilitates glucose metabolism by increasing the translocation of glucose transporters to the plasma membrane (Nicholson and Anderson, 2002). After glucose is transported inside cells, it can fuel mitochondrial respiration for ATP production during oxidative energy metabolism (Gonzalez-Lima et al., 2014). In this sense, the search for new therapies for the treatment or prevention of age-related harm is necessary for the medical society.

Transcranial stimulation with near-infrared light is a type of low–level laser therapy or photobiomodulation (PBM; Anders et al., 2015), which has been used recently as a non-pharmacological tool for the treatment of brain damage and age-related cognitive decline (Naeser et al., 2014; Vargas et al., 2017; Chan et al., 2019). For example, Naeser and collaborators (Naeser et al., 2014) demonstrated that 6 weeks of red/near-infrared light-emitting diode treatment improved the executive function and verbal memory of elderly people with chronic mild traumatic brain injury. Vargas and collaborators (Vargas et al., 2017) showed that a laser session with 1,064 nm wavelength and fluence of 60 J/cm^2^ per week for 5 weeks improved the cognitive function and EEG rhythms of older adults with memory complaint. Using D-galactose-induced aging mice, Salehpour and collaborators (Salehpour et al., 2017) noted that red (660 nm) and near-infrared (810 nm) laser for 6 weeks attenuated the impairment in spatial and episodic-like memories. One hypothesis to explain these effects may be related to the capacity of PBM to regulate neuronal functions (Eells et al., 2003; Rojas et al., 2008; Freitas and Hamblin, 2016), including cell proliferation (Shefer et al., 2002; Gao et al., 2006), DNA and protein synthesis (Feng et al., 2012), and oxidative energy metabolism (Wang et al., 2017b).

Interestingly, some studies have shown that PBM can alter the expression of intracellular signaling proteins in several conditions (Yip et al., 2011; Meng et al., 2013; Zhang et al., 2020). For example, Yip and collaborators (Yip et al., 2011) noted that only one laser session was able to increase the Akt activation in mice after transient cerebral ischemia. Zhang and collaborators (Zhang et al., 2020) observed an increase in activation of PKA and SIRT1 in beta amyloid protein precursor transgenic mice. Based on this, this study investigated the cellular signaling proteins in the cortex and hippocampus of aged rats (20 months old) subjected to either a PBM protocol or a placebo/sham control for 58 consecutive days. For testing brain PBM effects, we analyzed the activation and expression of intracellular signaling proteins, such as Akt, p70S6K, signal transducer and activator of transcription 3 (STAT3), signal transducer and activator of transcription 5 (STAT5), ERK, JNK, and p38.

## Materials and Methods

### Animals

Eleven male Wistar rats, aged (20 months old) were used in this study. The colony room was maintained at 21 ± 2°C with a 12 h light/ dark schedule (light: 7 am until 7 pm), and food and water were provided *ad libitum* throughout the experimental period. All experimental protocols were approved by the ethics committee of the University of Mogi das Cruzes (UMC; # 016/2017) and all efforts were made to minimize animal suffering in accordance with the proposals of the International Ethical Guideline for Biomedical Research (Council for International Organizations of Medical Sciences, 1985).

### Laser and Control Protocols

The rats were randomly distributed into two groups: laser (*n* = 6) and control (*n* = 5). One week before the treatment protocol, the animals were adapted to manual handling (with no anesthesia). This procedure was adopted to minimize discomfort during treatment. After this, the animals from the laser group were manually immobilized and received treatment with a laser diode of 810 nm wavelength and 100 mW power for 30 s (3 Joules of energy/point) by approximation at each of the 5 irradiation points of application (point 1 = AP +4.20 mm and ML 0.00 mm; point 2 = AP −3.00 mm and ML −6.60 mm; point 3 = AP −3.00 mm and ML +6.60 mm; point 4 = AP 0.00 mm and ML 0.00 mm; point 5 = AP −5.52 mm and ML 0.00 mm). The daily laser treatment total was 15 Joules of energy, 150 s of irradiation, and fluency of 535.7 Joules/cm^2^. Also, no difference in scalp temperature measured in the animals was noted with a non-contact thermometer during the treatment protocol, corroborating with the results of Wang and collaborators (Wang et al., 2017a). Animals were exposed to the transcranial low-level laser over 58 consecutive days. Our rationale was to investigate the therapeutic capacity of chronic treatment with laser. Animals from the control group received the same procedure as the laser group, but as placebo sham control (laser off). The parameters used in the present study were based on our previous studies with different animal models (Haslerud et al., 2017; Naterstad et al., 2018).

#### List of Laser Parameters

Center wavelength (nm): 810
Operating mode: CW
Average radiant power (W): 0.1
Aperture diameter (cm): 0.6
Irradiance at aperture (W/cm^2^): 3.571
Beam shape: Circular
Beam spot size (cm^2^): 0.028
Irradiance at target (W/cm^2^): 3.571
Exposure duration/point (s): 30
Radiant exposure (J/cm^2^) per session: 535.7
Number of points irradiated: Five
Delivery mode: contact mode
Number and frequency of sessions: one session/day for 58 consecutive days.
Total radiant energy (J) per head: 15

### Analysis of Intracellular Proteins Levels

#### Tissue Preparation

Twenty-four hours after the final laser/placebo session (59th day of the experiment), the rats were euthanized by decapitation and their cerebral cortex (all cortical tissue) and hippocampus (Ammon’s horn and dentate gyrus) were immediately collected and frozen. The tissue samples were homogenized in ice-cold RIPA lysis buffer (50 mM Tris-HCl, pH 7.5, 150 mM NaCl, 0.5% sodium deoxycholate, 1% NP-40, 0.1% SDS) with freshly added protease (Cat# M222–1ml; Lot# 1295C056; Amresco) and phosphatase (Cat# B15001-A and B; Lot# 510011; Biotool) inhibitors. Homogenates were centrifuged at 10,000× *g* for 10 min at 4°C and supernatants were transferred to a new tube.

#### Intracellular Proteins Measurements

The expression and activation (phosphorylated/total) of intracellular proteins in the brain samples were quantified using the Milliplex^®^ MAP kits magnetic bead panel assay (48-680MAG and 48-681MAG, Merck Millipore) following the manufacturer’s specifications. This multiplex immunoassay allows the simultaneous quantification of the expression and activation of cortical and hippocampal proteins: Akt, p70S6K, STAT3, STAT5, ERK, JNK, and p38. The plates were read on a Luminex^TM^ Magpix^TM^ instrument and results were analyzed with the Milliplex Analyst 5.1 Software using a Logistic 5P Weighted regression formula to calculate sample concentrations from the standard curves.

### Statistical Analysis

Statistical procedures were conducted using the Mann-Whitney U-Test for independent group comparisons of nonparametric data. The Z-score was used to remove outlier values (−/+2 SD). In the cortex, we removed one outlier of p-ERK/ ERK from the control group and one outlier of p-p38/p38 from the laser group. In the hippocampus, we removed one outlier of STAT3 and STAT5 from the control group and one outlier of p-ERK/ERK from the laser group. All analyses were performed using the Statistical Package for the Social Science (SPSS Inc, IBM, version 221.0, Chicago, IL, USA). A statistical difference was considered significant when the two-tailed *P*-value was lower than 0.05. All plots were acquired using the Graph Pad Prism (6.0). Data are presented as individual points to show dispersion and as mean and standard error of the mean (±SEM).

## Results

**Figure 1:**
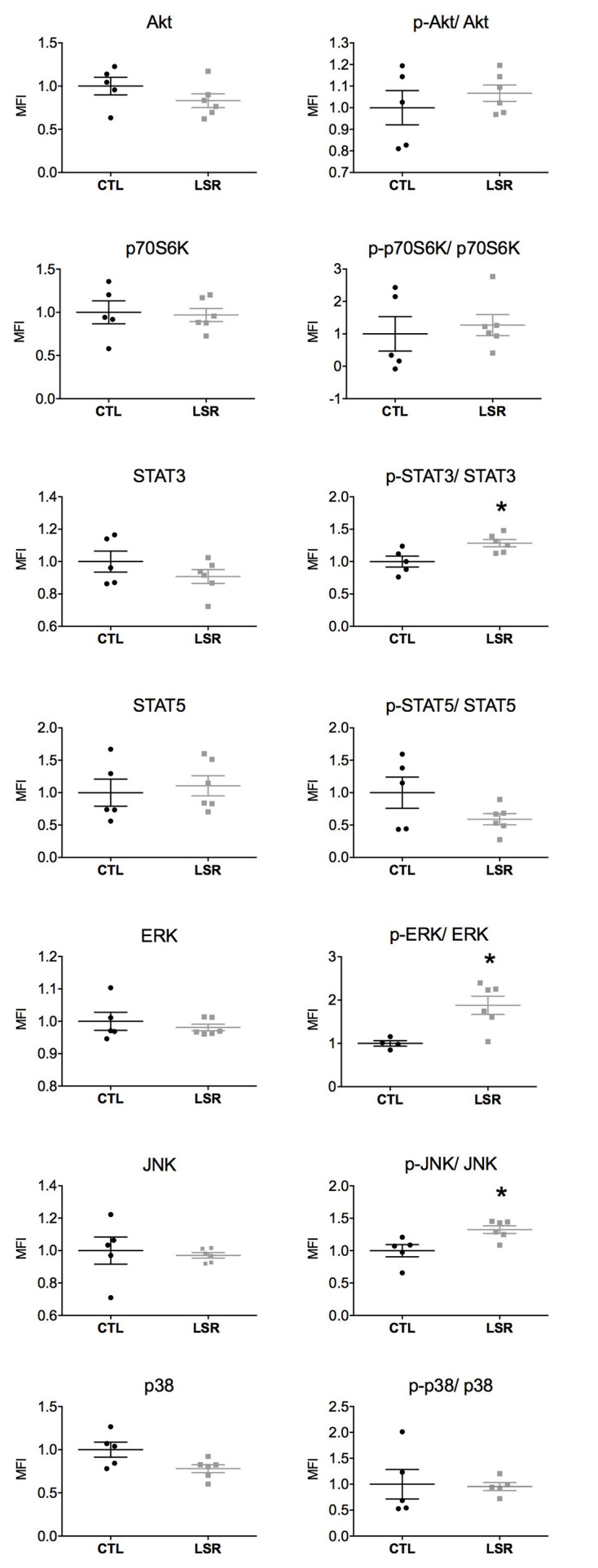
Cortical expression and activation (phosphorylated/total protein) of intracellular signaling pathways in rats from the laser (LSR) and control (CTL) groups. Data are presented as individual points to show dispersion and as mean and standard error of the mean (±SEM). Significant increases in the activation of STAT3, ERK, and JNK were found in the laser group when compared to the control group (*). Data were normalized to the mean fluorescence intensity (MFI) of the control group (*p* < 0.05; Mann-Whitney U-Test).

**Figure 2:**
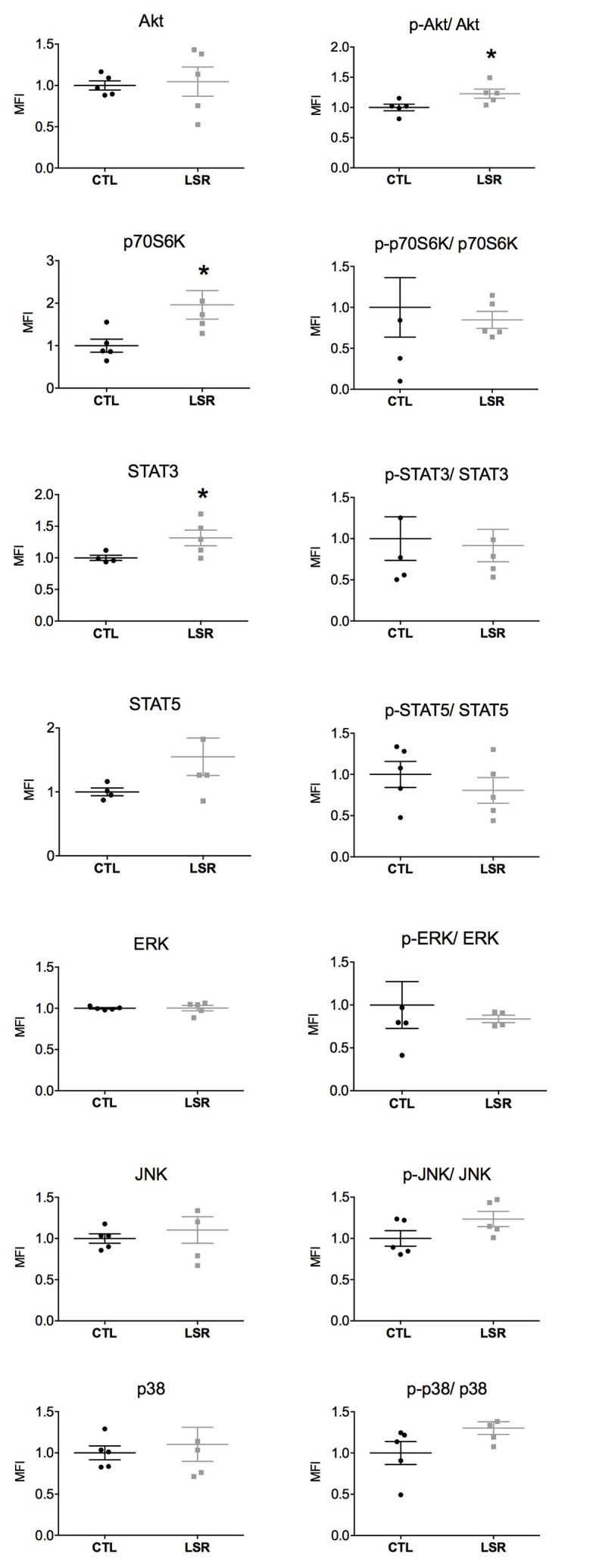
Hippocampal expression and activation (phosphorylated/total protein) of intracellular signaling pathways in rats from the laser (LSR) and control (CTL) groups. Data are presented as individual points to show dispersion and as mean and standard error of the mean (±SEM). Significant increases in the expression of p70S6K and STAT3 and Akt activation were found in the laser group when compared to the control group (*). Data were normalized to the mean fluorescence intensity (MFI) of the control group (*p* < 0.05; Mann-Whitney U-Test).

The expression and activation of intracellular signaling proteins (Akt, p70S6K, STAT3, STAT5, ERK, JNK, and p38) were investigated in the cerebral cortex and hippocampus of rats from the laser and control groups. The cortical and hippocampal expression and activation are presented in (Figures 1, 2). The figures showed that measures from laser-treated rats had less data dispersion than for the control rats for most proteins. However, data dispersion appeared similar for both cortex and hippocampus.

### Cortical Expression and Activation of Intracellular Signaling Pathways

No significant difference between the groups studied was observed in the cortical expression of Akt (*U* = 8.000; *p* = 0.201), p70S6K (*U* = 12.000, *p* = 0.584), STAT3 (*U* = 11.000; *p* = 0.465), STAT5 (*U* = 12.000; *p* = 0.584), ERK (*U* = 13.000; *p* = 0.715), JNK (*U* = 9.000; *p* = 0.273) and p38 (*U* = 5.000; *p* = 0.068). With regard to the activation of signaling proteins (phosphorylated/total), no significant difference was observed between the groups studied in the cortical activation of Akt (*U* = 12.000; *p* = 0.584), p70S6K (*U* = 10.000; *p* = 0.361), STAT5 (*U* = 10.000; *p* = 0.361) and p38 (*U* = 10.000; *p* = 0.602). However, PBM significantly increased the cortical activation of STAT3 (*U* = 2.000; *p* = 0.018), ERK (*U* = 1.000; *p* = 0.019) and JNK (*U* = 2.000; *p* = 0.018; Figure 1).

### Hippocampal Expression and Activation of Intracellular Signaling Pathways

No significant difference between the groups studied was observed in the hippocampal expression of Akt (*U* = 11.000; *p* = 0.754), STAT5 (*U* = 4.000; *p* = 0.140), ERK (*U* = 10.000; *p* = 0.602), JNK (*U* = 10.000; *p* = 0.602) and p38 (*U* = 12.500; *p* = 1.000). However, PBM significantly increased the hippocampal expression of p70S6K (*U* = 2.000; *p* = 0.028) and STAT3 (*U* = 1.000; *p* = 0.027; Figure 2). In relation to the activation of signaling proteins, no significant difference was observed between the groups studied in the hippocampal activation of p70S6K (*U* = 12.000; *p* = 0.917), STAT3 *U* = 12.000; *p* = 0.917), STAT5 (*U* = 4.000; *p* = 0.142) ERK (*U* = 8.000; *p* = 0.624), JNK (*U* = 6.000; *p* = 0.175) and p38 (*U* = 5.000; *p* = 0.117). However, PBM significantly increased the hippocampal activation of Akt (*U* = 2.000; *p* = 0.028; Figure 2). Taken together, these data show that PBM changes the expression and activation of intracellular signaling proteins in the brain of aged rats.

## Discussion

The purpose of our study was to investigate the cellular signaling proteins in the cortex and hippocampus of aged rats subjected to a PBM protocol with a laser of 810 nm wavelength and 100 mW power over 58 consecutive days.

In this study, our laser treatment increased the cortical activation of JNK in aged rats. However, we expected that the laser could reduce the JNK activation, as found in the aging mice study conducted by Salehpour and collaborators (Salehpour et al., 2019). This difference may be related to the different treatment protocols. They used a laser treatment protocol of 810 nm wavelength for five consecutive days (with a fluence of 33.3 J/cm^2^), while our protocol consisted of 58 consecutive days of laser treatment with 810 nm wavelength and fluence of 535.7 J/cm^2^. Therefore, our laser treatment was 10 times greater. PBM is well known to show hormetic dose-responses, with opposite effects at lower and higher doses (Rojas and Gonzalez-Lima, 2011; Xuan et al., 2013).

Furthermore, our laser treatment protocol also increased the cortical activation of ERK in aged rats. Activation of ERK and JNK signaling pathways play critical roles in regulating glucose metabolism (Papa et al., 2019). Improvement of glucose metabolism by PBM would be particularly beneficial to aging brains that are characterized by energy hypometabolism and memory decline (Gonzalez-Lima et al., 2014). Our results are consistent with the findings of Meng and collaborators (Meng et al., 2013). They found an increase in ERK activation after a 632.8 nm laser treatment protocol with fluences of 0.5, 1, 2, and 4 J/cm^2^ in neuronal cells. This finding is important since the activation of ERK is involved in cellular proliferation, differentiation, and migration (Kim and Choi, 2010). In addition, the reduction of the ERK signaling pathway has been associated with long-term potentiation deficits and memory impairment in aged rats (English and Sweatt, 1997; Jin et al., 2008). Possibly, this is because ERK activation occurs when Thr183 and Tyr185 residues are phosphorylated by MAPKK or MEK1 (Anderson et al., 1990; Crews et al., 1992). After its activation, ERK activates regulatory proteins and kinases, as well as transcription factors (Fox et al., 1998). For this reason, ERKs are presumably present in processes of memory and synaptic plasticity (Sweatt, 2004).

Our results show that PBM increased the cortical activation of STAT3. STAT3 can modulate the expression of the anti-apoptotic genes of the Bcl-2 family by regulating neuronal survival (Fukada et al., 1996). In addition, JAK2/STAT3 signaling is critical for increasing neuronal proliferation and differentiation in IL-6 treated cells (Müller et al., 2009; Cheng et al., 2011). During aging, a decrease in STAT3 levels is observed (Bazhanova and Anisimov, 2016).

In the hippocampus, our results showed that PBM increased the expression of p70S6K and STAT3 and activation of Akt in aged rats. Our data regarding activation of Akt corroborates the findings of Yip and collaborators (Yip et al., 2011) in mice after transient cerebral ischemia exposed to a laser treatment session with three different fluences (2.64, 13.20, or 26.40 J/cm^2^). These results suggest a neuroprotective effect of PBM since these proteins are linked to cell survival, apoptosis, and protein synthesis (Vara et al., 2004; Harrington et al., 2004; Wang and Proud, 2006), and in turn, they are decreased in the hippocampus of aged rats (Jin et al., 2008). For example, phosphatidylinositol 3-phosphate kinase (PI3K) activates Akt (Sheppard et al., 2012). Activated Akt is recruited to the cell membrane, promoting the phosphorylation of the residues: treonine-308 (Thr-308) and serine-473 (Ser-473; Alessi et al., 1996). Thus, Akt inhibits the pro-apoptotic pathways of the cell, such as the activity of the pro-apoptotic protein of the family Bcl-2 (Bad; Vara et al., 2004). Also, p70S6K is capable of inhibiting Bad protein (Harada et al., 2001). p70S6K signaling is linked to the activation of transcription and translation processes of essential genes for protein synthesis (Harrington et al., 2004; Wang and Proud, 2006). For example, p70S6K promotes an increase in synaptic transmission-related proteins, such as postsynaptic density protein-95 (PSD-95) and a-amino-3-hydroxy-5-methyl-4- isoxazolepropionic acid receptor (AMPAR; Lee et al., 2005; Hoeffer and Klann, 2010), contributing to the maturation of excitatory synapses (El-Husseini et al., 2000) and improving synaptic transmission and plasticity (Migaud et al., 1998; Ehrlich and Malinow, 2004).

Finally, PBM-induced activation of Akt can also contribute to enhancing the PBM-induced activation of ERK and JNK signaling pathways, which are crucial in programing glucose metabolism (Papa et al., 2019). That is because Akt increases the translocation of glucose transporters to the plasma membrane (Nicholson and Anderson, 2002), suggesting that PBM can promote energy metabolism in the aged brain *via* these intracellular signaling pathways.

A limitation of this study was the lack of a group of young animals to compare with aged animals. It would be interesting to investigate the effects of age on the expression and activation of intracellular signaling proteins investigated in this study. Also, we would be able to analyze whether the effects of laser treatment are different in young and aged rats. These analyses would provide more information on the therapeutic potential of PBM in age-related disorders. Despite this, the current study is the first to investigate the effects of PBM on intracellular signaling proteins in the brain of healthy aged rats. Recently, many authors have investigated the therapeutic potential of laser treatment in diseases related to brain aging (Meng et al., 2013; El Massri et al., 2017; Zhang et al., 2020). However, little is known about the effects of PBM on the old brain without the disease. Another important point is the inter-individual variability observed during aging (Nyberg, 2017; Myrum et al., 2019). However, our results corroborate studies using laser treatment in other tissues (Kim et al., 2017; Tani et al., 2018; Wang et al., 2021). Other limitations are the small number of rats used and the low output power and the very small spot size of the laser device used. Nevertheless, our research group has found interesting results in various tissues using this same laser device with similar numbers of rats (Haslerud et al., 2017; Naterstad et al., 2018; Cardoso et al., 2021).

Our data indicate that transcranial PBM improves intracellular signaling pathways linked to cell survival, memory, and glucose metabolism in the brain of aged rats.

## Data Availability Statement

The raw data supporting the conclusions of this article will be made available by the authors, without undue reservation.

## Ethics Statement

The animal study and all experimental protocols were reviewed and approved by the ethics committee of the University of Mogi das Cruzes (UMC; # 016/2017).

## Author Contributions

Conceived and designed the experiments: FC, FM, and SG. Performed the experiments: FC and FM. Analyzed the data: FC and SG. Contributed reagents, materials, and analysis tools: FC and SG. Wrote the manuscript: FC, RL-M, FG-L, and SG. Approved the final version of the manuscript: FC, FM, RL-M, FG-L, and SG.

## Conflict of Interest

The authors declare that the research was conducted in the absence of any commercial or financial relationships that could be construed as a potential conflict of interest.

## Publisher’s Note

All claims expressed in this article are solely those of the authors and do not necessarily represent those of their affiliated organizations, or those of the publisher, the editors and the reviewers. Any product that may be evaluated in this article, or claim that may be made by its manufacturer, is not guaranteed or endorsed by the publisher.
